# Cellular Uptake of Epigallocatechin Gallate in Comparison to Its Major Oxidation Products and Their Antioxidant Capacity In Vitro

**DOI:** 10.3390/antiox11091746

**Published:** 2022-09-02

**Authors:** Julian Alfke, Melanie Esselen

**Affiliations:** Institute of Food Chemistry, University of Münster, Corrensstr. 45, 48149 Münster, Germany

**Keywords:** human liver carcinoma HepG2 cells, theasinensins, oolongtheanin, cellular uptake, dichlorofluorescein

## Abstract

Depletion of reactive oxygen species and reduction of oxidative stress have been identified as key parameters in the prevention of cellular aging. In previous in vitro studies, the tea catechin epigallocatechin gallate (EGCG) was found to have both pro- and antioxidant properties, disregarding the low stability under cell culture conditions. Besides hydrogen peroxide, theasinensin dimers amongst other oxidation products are formed. Exact quantities, cellular uptake and antioxidant capacities of these dimeric oxidation products remain unknown. Via high-performance liquid chromatography (HPLC) coupled with tandem mass spectrometry (MS/MS), formation kinetics and cellular uptake of EGCG and its major oxidation products are quantified. The antioxidant capacity is determined on a cellular level using a modified dichlorofluorescein (DCF) approach. As a first result, oxidation product quantities of up to 21 µM each are measured after incubation of 50 µM EGCG. While EGCG is taken up equimolarly, its major oxidation products are accumulated in hepatocarcinoma HepG2 cells at millimolar concentrations, especially theasinensin A (TSA). Lastly, the oxidation products show higher antioxidant properties than the monomer EGCG. In correlation with cellular uptake, TSA displays the highest capacity of all tested analytes. The findings reveal the strong influence of EGCG oxidation products on its bioactivity in vitro.

## 1. Introduction

**Figure 1:**
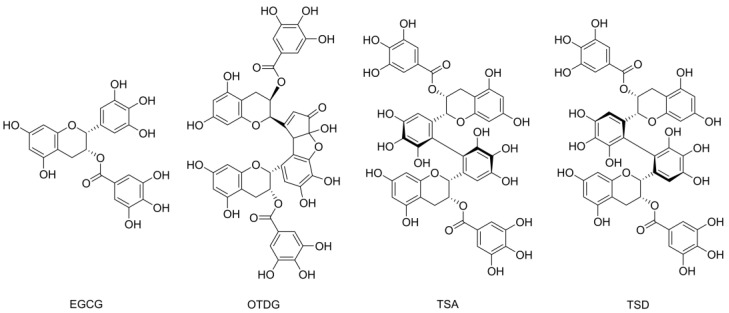
Chemical structures of the flavan-3-ol epigallocatechin gallate (EGCG) and its oxidation products oolongtheanin digallate (OTDG), theasinensin A (TSA) and theasinensin D (TSD).

The flavan-3-ol epigallocatechin-3-gallate (EGCG, see Figure 1) represents one of the most famous natural compounds with positive health-related bioactive connotation [1]. With tea being a popular beverage worldwide, its abundant flavonoid EGCG has been the compound of interest of many biochemical and pharmaceutical investigations [1,2]. While positive properties—for example, anti-inflammatory effects—have been found in vitro and in vivo, adverse attributes are also known [1,3,4]. EGCG hepatotoxicity has been described at higher exposure by the European Food Safety Authority (EFSA) in 2018 [5]. Regarding antioxidant properties, both pro- and antioxidant characteristics were determined [4,6,7]. EGCG can act as radical scavenger diminishing reactive oxygen species (ROS), but its chemical behavior limits its bioactivity [7,8]. Extremely low stability under cell culture conditions was observed in different media, and hydrogen peroxide—formed through EGCG degradation—is known to induce oxidative stress itself [9,10,11]. Therefore, EGCG-related in vitro effects can take many forms [7]. Previous cell-free studies on EGCG’s antioxidant capacity might not consider these crucial parameters.

Another aspect of EGCG’s properties under cell culture conditions is the formation of oxidation products. While there are plenty of already described dimers and oligomers on the basis of galloylated catechins, the theasinensins and oolongtheanin digallate (OTDG) represent major dimeric oxidation products of EGCG (see Figure 1) [12,13]. These compounds are not only formed in an autoxidative manner, but also occur after enzymatic oxidation, e.g., during tea fermentation [14]. With contents exceeding 40 mg/L in black tea infusions, theasinensin A (TSA) was observed especially, but theasinensin D (TSD) and OTDG also play a role in the exposure of tea consumers [15].

Due to the lack of EGCG stabilization in many cell culture studies regarding EGCG’s in vitro effects, it is hard to understand whether the described bioactive attributes are based on EGCG or its oxidation products [11,12,16]. In vitro theasinensin quantities after EGCG incubation could only be estimated. Furthermore, previous information on flavan-3-ol effects on various signaling pathways should be reviewed in the context of cellular uptake, which has not been elucidated so far. In fact, studies show antagonistic effects between EGCG and the theasinensins regarding cellular interaction [17]. Trans-membrane receptors such as 67 kDa lamin receptor (67-LR)—which is reported as high affinity EGCG receptor—might also be involved in the bioactivity of theasinensins and other EGCG oxidation products [18].

As the aim of this work, EGCG’s role as an abundant, antioxidant natural compound is scrutinized and evaluated focusing on stability and formation of theasinensins and other oxidation products under cell culture conditions. For this, formation and degradation kinetics of EGCG and major oxidation products are analyzed via HPLC-MS/MS, and the cellular uptake of these compounds in HepG2 liver carcinoma cells is quantified. Lastly, antioxidant capacity of these analytes is determined on a cellular level using a modified dichlorofluorescein approach. After statistical analysis, the antioxidant properties of EGCG and the major oxidation products present under cell culture conditions are compared.

## 2. Materials and Methods

### 2.1. Chemicals and Reagents

Each chemical and reagent used is given with the corresponding abbreviation, purity, manufacturer and city/country of origin (if not stated before). Pure water is generated by a miniRO reverse osmosis station (Veolia Water Solutions & Technologies ELGA GmbH, Celle, Germany). For incubation in cell culture experiments in general, EGCG (>99%, Extrasynthese, Genay, France) oxidation products TSA (>95%), TSD (>99%) and OTDG (>96%) are isolated and structurally elucidated based on a previously published protocol [13].

Test compound stabilization for cellular uptake and formation evaluation is carried out with l-ascorbic acid (≥99%, p. a., Carl Roth GmbH & Co. KG, Karlsruhe, Germany), catalase from bovine liver (2000–5000 U/mg protein, Sigma-Aldrich Chemie GmbH, Taufkirchen, Germany), and tripotassium phosphate (≥98%, Sigma-Aldrich). Additionally, sodium ethylenediaminetetraacetic acid (Na-EDTA, Carl Roth) is used for cell washing and stabilization.

Regarding cellular uptake, HepG2 hepatocarcinoma cells (DSMZ, German Collection of Microorganisms and Cell Cultures GmbH, Braunschweig, Germany) are cultivated with Dulbecco’s modified Eagle’s medium (DMEM, Fisher Scientific GmbH, Schwerte, Germany) with 1% penicillin/streptomycin solution (1/1 *v*/*v* 10,000 U/mL/10,000 µg/mL, Fisher Scientific) and 10% fetal calf serum (FCS, Fisher Scientific). Other materials in use for cellular uptake experiments and antioxidant capacity determination are PBS buffer containing sodium chloride (180 g, Carl Roth), potassium hydrogen phosphate (4.2 g, Carl Roth) and disodium phosphate (8.18 g, Carl Roth) in 1 L of pure water at pH 7.4, dilution with pure water 1 + 19 *v*/*v*; trypsin solution (0.05% *w*/*v*, Fisher Scientific), acetonitrile (ACN, LC-MS purity, Fisher Scientific) for cell extraction and protein precipitation and as solvent, and CASY ton buffer (OMNI Life Science GmbH & Co. KG, Bremen, Germany) for cell counting.

Chromatographic separation via HPLC is operated using pure water and methanol (MeOH, LC-MS purity, Fisher Scientific) with formic acid (FA, 98−100%, p. a., Merck KGaA, Darmstadt, Germany).

For the determination of antioxidant capacities, 2′,7′-dichlorofluorescein diacetate (DCFH_2_-DA, ≥97%, Sigma-Aldrich), *tert*-butyl hydroperoxide solution (TBH, 70% *w*/*v*, Sigma-Aldrich) and DMEM w/o phenol red (Fisher Scientific) are obtained for cell incubation and ROS induction.

### 2.2. HPLC-MS/MS Method Settings, Calibration, Validation and Data Handling

The quantification of medium and cell lysate samples is performed on a Bruker Elute HT HPLC pump and EVOQ Elite triple-quad mass spectrometer (both Bruker Daltonics GmbH & Co. KG, Bremen, Germany) with a PAL HTC-xt autosampler (CTC Analytics, Zwingen, Switzerland) and a Bruker Elute column oven. The method development is based on previous quantification experiments and is featured in a previously published study [15].

As a stationary phase for the chromatographic separation, a Nucleodur phenyl-hexyl column (50 mm × 2 mm × 3 µm, Macherey-Nagel, Düren, Germany) is utilized. At a constant flow rate of 400 µL/min and an oven temperature of 40 °C, the following gradient with MeOH (A) and pure water (B) with 0.1% FA each is applied: 0 min 5% A, 1 min 5% A, 8 min 40% A, 8.75 min 100% A, 10.5 min 100% A, 10.51 min 5% A, 12 min 5% A.

As mass spectrometry source parameters, the following settings are applied: MS valve in a divert position between 0–0.8 min; probe/nebulizer/cone gas flow 50/60/20 psi, respectively; probe/cone/manifold temperature 500/250/42 °C, respectively; collision gas pressure 1.5 mTorr argon, positive/negative spray voltage 5000/−4500 V, respectively; activated exhaust gas.

For tandem mass spectrometric determination, polarity, quantifier and qualifier transitions Q1→Q3, collision energies (CE), and scan times (ST) are optimized for each analyte; and given in list format: EGCG, polarity positive, quantifier transition/CE/ST *m*/*z* 459.1→139.0/12 eV/40 ms, qualifier transition/CE/ST *m*/*z* 459.1→151.1/7 eV/40 ms; OTDG, polarity negative, quantifier transition/CE/ST *m*/*z* 883.1→543.1/20 eV/60 ms, qualifier transition/CE/ST *m*/*z* 883.1→712.9/14 eV/60 ms; TSA, polarity negative, quantifier transition/CE/ST *m*/*z* 913.1→591.1/28 eV/40 ms, qualifier transition/CE/ST *m*/*z* 913.1→573.1/20 eV/40 ms; TSD, polarity negative, quantifier transition/CE/ST *m*/*z* 913.1→591.1/22 eV/40 ms, qualifier transition/CE/ST *m*/*z* 913.1→743.1/12 eV/40 ms.

Calibration of medium and cell lysate samples is performed using matrix-matched calibration techniques to ensure true quantification results and to minimize matrix effects. Therefore, two separate calibrations were measured each in triplicate, with respect to the varying sample preparation of medium supernatant samples and lysed cell extracts. Linearity of the calibration curves is ensured using Mandel’s fitting test. Method validation parameters are recorded and calculated, involving limit of determination (LOD) and limit of quantification (LOQ), recovery rates and repeatability values, and given in the previous publication [15].

### 2.3. Formation of EGCG Oxidation Products under Cell-Free Cell Culture Conditions

The formation of EGCG oxidation products is analyzed through incubation of 50 µM EGCG in serum-free DMEM without supplemented ascorbic acid and catalase. The solution is directly heated up to 37 °C in a micro-reaction incubator. For time points up to 32 min, samples are collected, directly diluted with stabilization solution 1 + 9 *v*/*v* as described before and cooled to 4 °C. Samples are directly analyzed via HPLC-MS/MS, and each medium incubation experiment is performed in triplicate as *n* = 3.

### 2.4. Determination of HepG2 Cellular Uptake

For the cellular uptake determination, 900,000 HepG2 cells are seeded into 60 mm cell culture dishes and allowed to grow in 5 mL FCS-DMEM at 37 °C and 5% CO_2_ for 48 h in total. After 24 h, the culture medium is changed to serum-free conditions. Another 24 h later, the medium is removed, and the test substances are incubated in fresh, serum-free DMEM supplemented with 1 mM ascorbic acid (prepared in pure water) and 500 U/mL catalase (prepared in 50 mM phosphate buffer at pH 7.0). Samples are drawn directly after incubation (medium only) and after 3 h, 6 h, 9 h and 12 h (both medium and cell lysate). Each cell incubation experiment is performed in triplicate as *n* = 3. As stabilization solution for both sample preparation methods, a solution of 500 µg/mL ascorbic acid, 500 µg/mL Na-EDTA and 5% MeOH *v*/*v* in pure water is prepared.

Medium aliquot samples are diluted 1 + 4 *v*/*v* with ACN (−20 °C), vortexed and centrifugated at 15,000× *g* for 4 min at 4 °C. The supernatant is diluted 1 + 9 *v*/*v* with stabilization solution as mentioned before. The samples are stored at 4 °C and directly analyzed via HPLC-MS/MS.

For the analysis of cell lysates, the medium is removed, and cells are washed with 3 mL 0.02% *w*/*v* Na-EDTA. After cell detachment with 2 mL trypsin solution for 4 min at 37 °C, the cell suspension is transferred into a tube. The cell culture dish is washed and rewashed with 2 × 4 mL PBS in the same tube, resulting in a cell suspension with an end volume of 10 mL. Using an aliquot of this suspension with a cell counting system (CASY Model TT, OMNI Life Science), both the absolute cell count and the cellular volume are measured. The cell suspension is centrifugated at 860× *g* for 5 min at 4 °C, and the supernatant is discarded. In addition, 1 mL of ACN/pure water 4 + 1 *v*/*v* is put onto the precipitate, and the tube is vortexed, followed by lysis in an ultrasonic bath for 15 min. After centrifugation at 860 g for 5 min at 4 °C, the supernatant is quantitatively transferred into a glass vial, in which the liquid phase is evaporated using constant nitrogen flow at 30 °C. The resulting solid residue is reconstituted with 500 µL stabilization solution, vortexed thoroughly, and stored at 4 °C until HPLC-MS/MS analysis. For the calculation of cellular concentrations, the concentration during sample preparation as well as the measured cellular volumes are considered.

### 2.5. Evaluation of Antioxidant Capacity In Vitro Using a Modified Dichlorofluoresceine Diacetate (DCFH_2_-DA) Assay

In a black, cell-compatible 96 well plate, 12,000 HepG2 cells/well are seeded in 200 µL FCS-DMEM each and allowed to grow for 72 h at 37 °C and 5% CO_2_. The outer rows and columns of the well plate are filled, but not used later to ensure comparable conditions throughout the well plate, leaving 60 remaining wells in the middle. The cells are washed with 200 µL/well PBS and afterwards pre-treated with the respective test analyte in different concentrations. For every well, a 1 + 99 *v*/*v* dilution of test analyte solution (or in the case of the negative and positive control ACN itself) in serum- and supplement-free DMEM takes place. The pre-incubation is carried out for 1 h under cell culture conditions. Ascorbic acid cannot be used for analyte stabilization due to its effect on the redox state of the cells. After removal of the pre-incubation media, the cells are washed with PBS as featured before. All the following steps are performed in the dark. Per well, 200 µL of 50 µM DCFH_2_-DA solution in PBS are added, and the well plate is incubated under cell culture conditions. After 30 min, the solution is removed, and the cells are washed with PBS as already described. In the last incubation step, 200 µL/well supplement-free DMEM without phenol red is put onto the negative control, while all the other wells—both positive control and pre-incubated test analyte—are incubated with 200 µL/well 250 µM TBH solution in supplement-free DMEM without phenol red. The well plate is directly placed in a microplate reader, shaken and fluorescence intensity is measured every 10 min for 2 h in total at λ_exc._ = 685 nm and λ_emi._ = 735 nm.

For the determination of the cellular redox status of each well, the difference in fluorescence between t_0_ and after 10, 30, 60 and 120 min is divided by the starting fluorescence t_0_. This relative fluorescence intensity increase is lastly related to the negative control. Each incubation experiment is performed in triplicate with two technical repetitions each as *n* = 3 × 2 = 6.

### 2.6. Software

For the handling of HPLC-MS/MS data and the calculation of peak areas, TASQ 2.2 (Bruker Daltonics GmbH & Co. KG) is used. Plotting of calculated data is performed with OriginPro 2021b. Microsoft Office 2019 is used for the calculation of quantification values and the antioxidant capacity, and the statistical evaluation using Student’s *t* test.

## 3. Results

**Figure 2:**
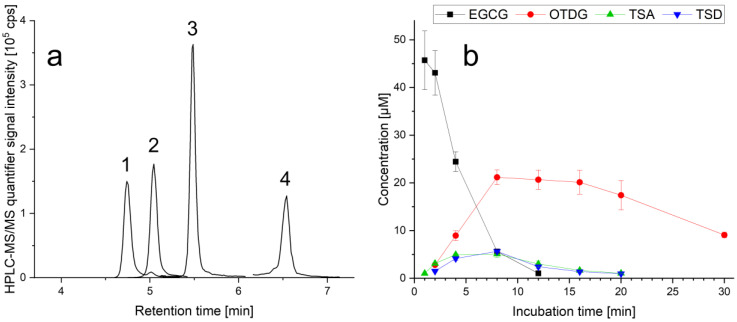
(**a**) Reconstructed HPLC-MS/MS chromatogram after method optimization, each as quantifier transition, labeled signals: 1: theasinensin A (TSA, 4 µg/mL), 2: epigallocatechin gallate (EGCG, 30 ng/mL), 3: theasinensin D (TSD, 4 µg/mL), 4: oolongtheanin digallate (OTDG, 4 µg/mL); (**b**) formation kinetics of the oxidation products OTDG, TSA and TSD after dilution of EGCG (50 µM) into single supplement-free DMEM cell culture medium at 37 °C, quantified via HPLC-MS/MS, *n* = 3.

The low stability of EGCG under cell culture conditions has been intensively described in literature and it questions the relevance of previous toxicological studies which do not include EGCG stabilization techniques. Emerging questions concerning the fate of EGCG in cell culture experiments have been answered by autoxidation reactions, especially at slight alkaline pH values. Nonetheless, information about the most abundant EGCG oxidation products is rare, and data on formation, cellular uptake and antioxidant properties of these compounds are still lacking. For the investigations on formation and cellular uptake of EGCG-related oxidation products, a universal quantification method via HPLC-MS/MS is developed, optimized and validated. In Figure 2a, a HPLC-MS/MS chromatogram of EGCG and its oxidations products TSA, TSD and OTDG is shown, demonstrating the chromatographic separation of the atropisomers TSA and TSD—obligatory for the simultaneous quantification of both dimers.

### 3.1. Stability of EGCG and Formation of EGCG Oxidation Products under Cell-Free Cell Culture Conditions

While the stability of EGCG and the formation of its oxidation products is dependent on several factors like temperature, pH value, dissolved oxygen levels and dual charged cations, cell culture experiments tend to feature harsh overall conditions for flavonoids. The formation of TSA, TSD and OTDG after incubation with EGCG was already observed in vitro [9,12,13]. To characterize exact formation kinetics of these products, a cell-free incubation experiment with standardized DMEM cell culture medium is executed to quantify the generated compounds via HPLC-MS/MS. In Figure 2b, the concentration course of EGCG as well as TSA, TSD and OTDG is shown after the incubation of supplement-free DMEM with 50 µM of non-stabilized EGCG at 37 °C, in relation to published toxicological in vitro studies regarding cytotoxicity [15].

As a parent compound, the concentration of EGCG rapidly decreases reaching levels below LOQ less than 16 min after incubation. The overall EGCG half-life t_1/2_ is observed to be less than 4 min. The EGCG dimers TSA and TSD are formed similarly, both reaching their highest medium concentrations after 8 min with 5.07 ± 0.69 µM and 5.63 ± 0.15 µM, respectively. As the third oxidation product, OTDG also scores its highest medium concentration after 8 min with 21.17 ± 1.54 µM. While the content of theasinensins is rapidly decreasing after reaching their maximum, OTDG concentrations show a plateau between 8 and 16 min, indicating a slower degradation and/or polymerization in comparison to TSA and TSD.

### 3.2. Cellular Uptake of EGCG and EGCG Oxidation Products in HepG2 Cells

**Figure 3:**
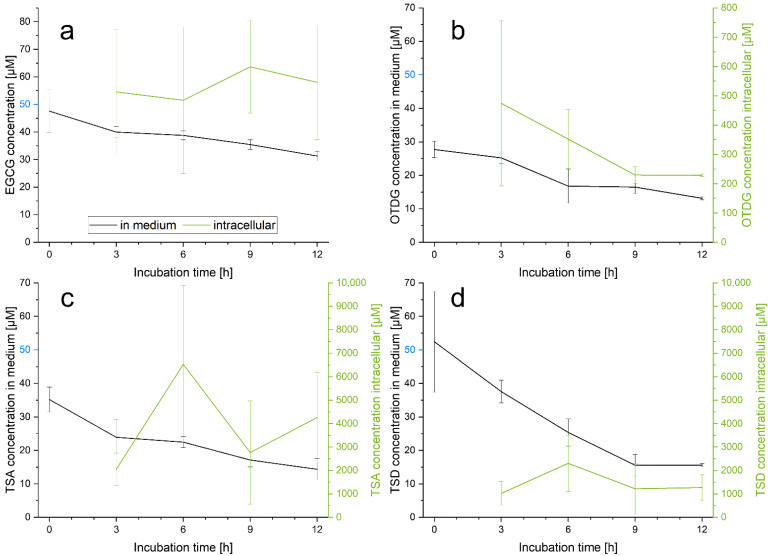
Cellular uptake assessment in HepG2 cells under stabilized conditions (see Section 2.4) quantified in medium and intracellularly as cell lysate via HPLC-MS/MS, *n* = 3, after incubation (marked in blue) of (**a**) EGCG, 50 µM; (**b**) OTDG, 50 µM; (**c**) TSA, 50 µM; (**d**) TSD, 50 µM.

The presence of EGCG oxidation products after incubation of EGCG in vitro raises questions regarding their cellular uptake. In consideration of the already observed cytotoxic effects of EGCG and the theasinensins on HepG2 liver carcinoma cells, their cellular uptake as well as their intracellular behavior and accumulation under stabilized conditions was analyzed via HPLC-MS/MS. In Figure 3a–d, the concentration of EGCG, TSA, TSD and OTDG both in medium and in cells is shown after treatment of HepG2 cells with 50 µM of each analyte up to 12 h.

First of all, the stabilizing effect of the supplement ascorbic acid and the cells themselves on the analytes is clearly observable, considering their slower degradation and oxidation—especially in comparison to the supplement- and cell-free experiment (see Section 3.1). For all compounds, even the lowest quantified concentration in medium is higher than 13 µM. In the case of EGCG, the stabilization leads to a prolonged stability under cell culture conditions. This demonstrates the benefit of the stabilization protocol with ascorbic acid; however, an impact of ascorbic acid on cellular metabolism cannot be excluded.

The initial levels of EGCG and its oxidation products for the analysis of intracellular concentrations are marked in blue in Figure 3. EGCG intracellular levels after 50 µM incubation (see Figure 3a) are nearly constant between 3–12 h. At every probed time point, the intracellular content fluctuates around the incubated concentration of 50 µM. Therefore, EGCG is taken up into HepG2 cells at equimolar amounts. Regarding OTDG, TSA and TSD, cellular uptake and transport through cell membranes seem to behave differently: The three analyzed oxidation products accumulate in HepG2 cells and their intracellular concentrations exceed the starting condition of 50 µM. For OTDG, intracellular amounts up to 474 µM are detected, passing over the starting concentrations by nearly factor 10 after 3 h. The highest intracellular level of all oxidation products is measured after TSA incubation, reaching a mean content up to 6529 µM after 6 h. Displaying a similar time course at overall lower concentrations, TSD is quantified with 2305 µM after 6 h, intracellularly.

### 3.3. Antioxidant Capacity of EGCG and EGCG Oxidation Products In Vitro

**Figure 4:**
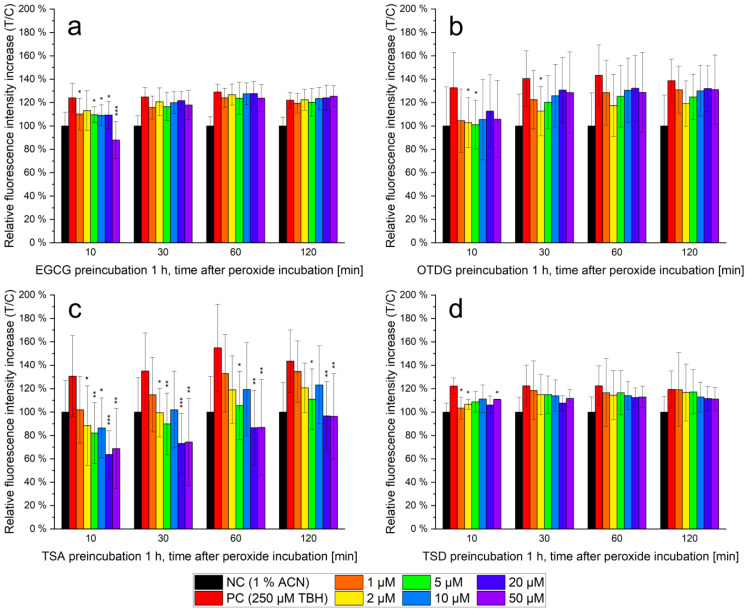
Modified DCFH_2_-DA assay for evaluation of antioxidant properties in vitro; relative fluorescence intensity increases in HepG2 cells after administration of DCFH_2_-DA and—except for the negative control (NC)—250 µM *tert*-butyl hydroperoxide (TBH), *n* = 6, statistical evaluation via Student’s two-way *t* test against the negative control with *P* as significance value marked with asterisks (* *p* = 95%, ** *p* = 99%, *** *p* = 99.9%), including a pre-incubation (except NC and positive control (PC)) with (**a**) EGCG, 1–50 µM; (**b**) OTDG, 1–50 µM; (**c**) TSA, 1–50 µM; (**d**) TSD, 1–50 µM.

The antioxidant capacity of compounds can be evaluated using a variety of techniques. While some methods rely solely on the chemical reductive properties—e.g., the Trolox equivalent antioxidant capacity (TEAC) assay—other approaches highlight antioxidant characteristics in vitro or in vivo. The modified DCFH_2_-DA assay can show antioxidant properties on a cellular level using DCFH_2_-DA as fluorophore that is liberated and therefore reactive only after cellular uptake. For the modified procedure according to Chen et al. and Bellion et al., cells are pre-incubated with the test analyte, followed by the administration of DCFH_2_-DA and TBH to induce intracellular ROS [19,20]. A time-dependent decrease of the TBH-induced relative fluorescence indicates antioxidant properties through intracellular ROS depletion by the test compound. Results are always described in comparison to the respective positive control (incubation with TBH without pre-incubation). Relative fluorescence values are given for 10, 30, 60 and 120 min after TBH incubation in Figure 4; test compound pre-incubation is performed between 1–50 µM for 1 h.

All tested compounds—EGCG, OTDG, TSA and TSD—show the highest antioxidant properties at low TBH incubation durations, especially after 10 min. With time progressing, relative fluorescence of the PC increases and the test analytes more and more approximate each other.

Considering EGCG (see Figure 4a) as parental flavan-3-ol, a concentration-dependent antioxidant effect is only observable after 10 min of peroxide incubation. In contrast, the relative fluorescence after 30, 60 and 120 min of peroxide incubation is increased to the level of the positive control. High antioxidant effects can be observed at TSA (see Figure 4c) over the whole incubation time up to 120 min. These effects are also concentration-dependent and statistically significant values are confirmed via a two-way Student’s *t*-test. For TSA, after 10 min and 30 min of peroxide treatment, ROS-induced fluorescence increases were lowered from 140% down to 60%, indicating an intense antioxidant effect of the preincubated analyte. Antioxidant activity of TSA is lowered after a prolonged treatment with TBH; however, at concentrations of 5 µM, the effect continues to be statistically significant. The oxidation products OTDG and TSD (see Figure 4b,d) behave similarly to EGCG: After 10 min of peroxide incubation, both show concentration-dependent antioxidant properties in vitro, with the highest decline in relative fluorescence increase at 50 µM OTDG or TSD, respectively.

## 4. Discussion

### 4.1. Stability of EGCG and Formation of EGCG Oxidation Products under Cell-Free Cell Culture Conditions

While many studies describe the low stability of EGCG under cell culture conditions, information on oxidation product contents after incubation of EGCG is lacking. For the evaluation of bioactive properties, it is most important to know which compounds are present, and especially at which concentrations. Therefore, levels of the most abundant oxidation products OTDG, TSA and TSD were quantified.

Regarding EGCG stability in cell culture medium at 37 °C, our study calculated EGCG’s half-life with 4 min in DMEM, with its complete degradation after 16 min. In contrast, Hong et al. determined EGCG degradation kinetics in McCoy’s 5A medium via radioflow detection. With a starting concentration of 10 µM, less than 10% of remaining analyte was detected after 1 h of dilution in medium. EGCG half-life in cell-free McCoy’s 5A medium is described with less than 30 min [9]. While the authors detected oolongtheanin and theasinensins—without their further separation—, a quantitation was not performed. Similarly, in cell-free Ham’s F12/RPMI-1640 medium (1/1 *v*/*v*), EGCG half-life is also calculated as <30 min, with EGCG being non-detectable after 6 h of medium dilution [12]. Here, all three of the oxidation products were determined without further quantitation. Regarding EGCG’s epimer gallocatechin gallate (GCG), stability in DMEM/Ham’s F12 medium (1/1 *v*/*v*) is also observed to be low, and with a starting concentration of 20 µM, the analyte could only be detected for 1 h after dilution [10]. These studies show the overall low stability of non-stabilized GCG and EGCG in cell-free media in line with results for the present findings. Nevertheless, oxidation product concentrations after EGCG incubation were not assessed before, and are now elucidated in DMEM cell culture medium. Concerning in vivo stability of EGCG and its autoxidation, slightly alkaline pH values are the basis of the formation of oxidation products like theasinensins [13]. These can be found in human blood, serum and saliva; therefore, the formation of EGCG oxidation products after uptake of EGCG is likely. Nevertheless, bioavailabilities need to be considered.

In summary, the formation experiment in single supplement-free DMEM demonstrates the significance of EGCG stabilization for biological evaluation. Even though studies show a stabilizing effect of cells on flavonoids in in vitro experiments, preparational dilution steps in cell-free medium are part of many standard operating procedures. The depicted stability data show that cell-free dilutions in medium must be avoided and EGCG stabilization techniques should be applied. Furthermore, the relevance of EGCG oxidation products to EGCG’s bioactivity is given, especially to estimate the influence of theasinensins and OTDG on previous findings in literature.

### 4.2. Cellular Uptake of EGCG and EGCG Oxidation Products in HepG2 Cells

Data availability on the cellular uptake of EGCG and especially its oxidation products is limited. Concerning HT29 cells, Hong et al. describe a time- and concentration-dependent uptake of EGCG until 3 h after incubation. The cytosolic concentration hereby exceeds the membrane-associated EGCG content [9]. Other studies suggest passive diffusion as cellular uptake transport mechanism of EGCG [9,17,21]. Efflux pumps and multidrug resistance-related proteins—MRP1 and MRP2—are observed to play crucial roles in the regulation of cellular EGCG levels [17,21]. As cellular EGCG concentrations equal to the starting concentration were found (see Figure 3a), a diffusional uptake of EGCG related to literature is suggested [17]. Nevertheless, an interaction between theasinensins and catechin-sensitive receptors is possible and should be further investigated. In addition to in vivo stability data, information on theasinensin and oxidation product bioavailabilities should be collected and considered for the evaluation of potential bioactivity.

For EGCG oxidation products, our study shows a vast accumulation in HepG2 cells. While comparable studies regarding this cell line do not exist, Neilson et al. found high rates of theasinensin and oolongtheanin uptake in human colon carcinoma Caco-2 cells, especially in comparison to flavan-3-ol monomers. The cellular uptake of oxidation products and dimers exceeded monomer uptake by factor 8 after 3 h of incubation [22]. In addition to these data, Luo et al. analyzed cellular uptake via viability experiments and found statistically decreased cell activities through EGCG incubation at 5 mg/mL and higher [23]. The described results for both colon HT29 and Caco-2 cell lines suggest that EGCG oxidation products from foods like tea infusions are likely to be absorbed in the gut. In tea infusions as a food matrix, theasinensin concentrations up to 4.13 mg/100 mL can be found [15]. In this context, interactions between EGCG, theasinensins and other oligomeric tea catechins are observed: The presence of TSA significantly decreases EGCG uptake in HT29 cells, which is explained by membrane intercalation of theasinensins [17]. This might affect the influx of EGCG into the cells and must be considered for the evaluation of flavanol-rich food matrices.

Other studies indicate a stronger cellular uptake of EGCG through the use of carrier nanoparticles [24,25]. In addition, liposomes can be superficially modified to increase EGCG cellular uptake [23,26].

The difference in cellular uptake between EGCG as monomer and its oxidation products enables the explanation of higher oxidation product cell cytotoxicity in the same cell line, as stated earlier [15]. Higher uptake might induce more severe biological effects through the influence of oxidation products on multiple signaling pathways inside the cell, including apoptosis. With the current data availability, theasinensin influence has been investigated on selected pathways only, and protective properties have been described [27,28,29,30,31].

### 4.3. Antioxidant Capacity of EGCG and EGCG Oxidation Products In Vitro

In several cell-free studies, the antioxidant properties of EGCG and other flavonoids were identified. These are usually explained structurally, especially regarding the vicinal hydroxy groups bound to the B ring of the compounds (see Figure 1). For an antioxidant effect in vitro, this capacity is analyzed on a cellular level, respecting all additional cell culture conditions which could influence compound antioxidant properties. The DCFH_2_-DA assay in its modified form was chosen because it allows the distinct analysis of antioxidant capacity and is highly fitting to the culture conditions and protocols of the cell line in use [19,32]. In addition, the determination of TBH-induced ROS in cells after pretreatment has been featured for similar experiments in literature [20].

The results of the modified DCFH_2_-DA assay (see Figure 4) show intracellular antioxidant capacities for EGCG and all three analyzed oxidation products, although to different extents. TSA features the highest intracellular concentration after incubation and also represents the compound with the highest antioxidant effect. In line with the above-mentioned results, the lowest antioxidant capacity is found with EGCG, the least abundant intracellular analyte. Therefore, a strong correlation between cellular uptake and intracellular antioxidant properties is suggested.

The results show that the antioxidant capacity of the compounds is not stable throughout the duration of 2 h after peroxide treatment: During the analysis, it decreases. Oxidative environments, which are to be found under cell culture conditions, lead to oxidation and consumption of antioxidants in solution. For flavonoids and especially EGCG, the high scavenging properties against ROS are described [33,34]. EGCG degradation after oxidation usually leads to dimeric or oligomeric oxidation products, but also features the formation of hydrogen peroxide [10,11,16]. These properties, the overall instability and the formation of hydrogen peroxide lead to the description of EGCG being a double-edged sword with both pro- and anti-oxidative attributes [7]. Due to the absence of ascorbic acid in this experimental setup—which is normally used to stabilize EGCG or its oxidation products in cell culture medium [10,15]—their stabilities are low (see Figure 2b). Nevertheless, the amount of substance after cellular uptake is sufficient to display the antioxidant properties intracellularly.

In comparison to previous, cell-free studies, in this present study, the antioxidant property of TSA seems considerably higher. The already mentioned TEAC assay relies on a comparable principle but is cell-free. Former TEAC assay results show the antioxidant capacity in the order TSD > TSA > OTDG [13]. Still, the three oxidation products were observed to be more antioxidative than EGCG by at least factor 1.54 [13]. Another study, based on the long-term oxidation of linoleic acid at increased temperature, did not identify differences in antioxidant capacity between EGCG and its oxidation products [35]. An explanation for this might be a faulty approach: the five-day incubation at 40 °C disregards the low stability of these compounds in these conditions [12,36].

Differences between the results of the previous cell-free TEAC assay and the DCFH_2_-DA assay in this publication can be explained via the varying cellular uptake of the compounds. While TSD was suggested to be the most antioxidant oxidation product [13], TSA’s high capacity in vitro might rely on its high abundance on a cellular level. In contrast to former experiments in literature, the DCFH_2_-DA assay takes physiological processes into account and draws a more realistic image of the intracellular situation. Furthermore, all antioxidant assays depict the oxidation products TSA and TSD with strong antioxidant capacities and are therefore consistent in the evaluation of EGCG dimers in comparison to its flavanol monomer. Therefore, the results of the cell-free TEAC assay and this cell-based approach do not contradict each other, but they answer different questions.

## 5. Conclusions

In summary, this study contributes new insights on the formation of EGCG oxidation products under cell culture conditions, their interaction with liver carcinoma cells and, especially, their antioxidant properties on a cellular level. While EGCG was shown to be instable in cell-free DMEM, oxidation product concentrations were quantified via HPLC-MS/MS. This enables the evaluation of past toxicological findings, if EGCG stabilization protocols have been missing. Cellular uptake of EGCG, TSA and TSD as well as OTDG in HepG2 cells indicates a vast intracellular accumulation of oxidation products. Lastly, the modified DCFH_2_-DA assay was used to characterize their antioxidant properties in vitro. In this context, the highest capacity was detected after TSA treatment, which strongly correlates with its cellular uptake on a millimolar level. All in all, EGCG and its oxidation products represent cell-penetrating flavan-3-ols with high antioxidant properties; thus, a contribution to the widely reported health effects of tea consumption is suggested.

## Data Availability

Not applicable.
